# Combined QTL and Genome Scan Analyses With the Help of 2b-RAD Identify Growth-Associated Genetic Markers in a New Fast-Growing Carp Strain

**DOI:** 10.3389/fgene.2018.00592

**Published:** 2018-12-07

**Authors:** Shengyan Su, Hengde Li, Fukuan Du, Chengfeng Zhang, Xinyuan Li, Xiaojun Jing, Liyue Liu, Zhixun Li, Xingli Yang, Pao Xu, Xinhua Yuan, Jian Zhu, Raouf Bouzoualegh

**Affiliations:** ^1^Key Laboratory of Freshwater Fisheries and Germplasm Resources Utilization, Ministry of Agriculture, Freshwater Fisheries Research Center, Chinese Academy of Fishery Sciences, Wuxi, China; ^2^Wuxi Fisheries College, Nanjing Agricultural University, Wuxi, China; ^3^Ministry of Agriculture Key Laboratory of Aquatic Genomics, CAFS Key Laboratory of Aquatic Genomics and Beijing Key Laboratory of Fishery Biotechnology, Center for Applied Aquatic Genomics, Chinese Academy of Fishery Sciences, Beijing, China; ^4^China Zebrafish Resource Center, Wuhan, China; ^5^Henan Academy of Fishery Sciences, Zhengzhou, China

**Keywords:** huanghe carp, 2b-RAD, Fst, growth traits, *igf2*

## Abstract

Common carp is one of the oldest and most popular cultured freshwater fish species both globally and in China. In a previous study, we used a carp strain with a long breeding tradition in China, named Huanghe, to create a new fast-growing strain by selection for fast growth for 6 years. The growth performance at 8 months of age has been improved by 20.84%. To achieve this, we combined the best linear unbiased prediction with marker-assisted selection techniques. Recent progress in genome-wide association studies and genomic selection in livestock breeding inspired common carp breeders to consider genome-based breeding approaches. In this study, we developed a 2b-RAD sequence assay as a means of investigating the quantitative trait loci in common carp. A total of 4,953,017,786 clean reads were generated for 250 specimens (average reads/specimen = 19,812,071) with BsaXI Restriction Enzyme. From these, 56,663 SNPs were identified, covering 50 chromosomes and 3,377 scaffolds. Principal component analysis indicated that selection and control groups are relatively clearly distinct. Top 1% of *Fst* values was selected as the threshold signature of artificial selection. Among the 244 identified loci, genes associated with sex-related factors and nutritional metabolism (especially fat metabolism) were annotated. Eighteen QTL were associated with growth parameters. Body length at 3 months of age and body weight (both at 3 and 8 months) were controlled by polygenic effects, but body size (length, depth, width) at 8 months of age was controlled mainly by several loci with major effects. Importantly, a single shared QTL (*IGF2* gene) partially controlled the body length, depth, and width. By merging the above results, we concluded that mainly the genes related to neural pathways, sex and fatty acid metabolism contributed to the improved growth performance of the new Huanghe carp strain. These findings are one of the first investigations into the potential use of genomic selection in the breeding of common carp. Moreover, our results show that combining the *Fst*, QTL mapping and CRISPR–Cas9 methods can be an effective way to identify important novel candidate molecular markers in economic breeding programs.

## Introduction

With the development of high throughput genotyping technology, the cost of genome-wide genetic markers decreased sharply, facilitating genomic selection (GS) and prediction of complex, quantitative traits (Meuwissen et al., 2001) After the first large-scale implementation of GS in dairy cattle breeding (Schaeffer, 2006), this technique has also been applied in other cultured species, such as pig (Knol et al., 2016), lupin (Yang et al., 2015), wheat (Charmet and Storlie, 2012), and Atlantic salmon (An et al., 2012; Correa et al., 2015; Tsai et al., 2015; Bangera et al., 2017). Common carp *Cyprinus carpio* L. is among the handful of oldest and most important cultured fish species, both globally (FAO, 2015) and in China, where it was ranked third among the cultured freshwater fish species in 2016, with the annual production of 349,800 tones (FFABMA, 2017). As a result of centuries-long breeding tradition, a strain of common carp named Huanghe is deeply rooted in Chinese culture, and remains popular among consumers for its cultural and historical relevance, although its growth performance is objectively somewhat worse than that of other modern strains (Zhang et al., 2013). Using this strain as the base, recently we established a new fast-growing strain (Su et al., 2018), here provisionally named *Xinlong* strain. The strain was selected for growth performance for 6 years using the best linear unbiased prediction (BLUP) and marker-assisted technology, resulting in a 20.84% improved growth rate of the F3 generation at 8 months of age. However, the genetics underlying this phenotypic difference remains unknown. Among these two breeding methods, BLUP can provide a higher genetic gain under controlled inbreeding rate and make full use of systematic effects (e.g., batch, sex, production environment, age variation, etc.), which can be estimated in a fitted mixed model for traits of interest (Ponzoni et al., 2010; Lind et al., 2012). As a result, BLUP is widely used in fish breeding (Lind et al., 2012).

Xu et al. published a draft genome of the domesticated common carp (strain Songpu), developed the first high-throughput array for 250,000 SNPs, and evaluated its performance using samples from various carp strains (Xu et al., 2014a). However, identification of candidate genes or loci for GS with a large number of SNPs remained costly (Xu et al., 2014b). To address this issue, restriction site–associated DNA (RAD) tag sequencing was developed, in which only the stretches of DNA adjacent to recognition sites of a chosen restriction endonuclease are resequenced (Davey et al., 2011). In order to further reduce the cost for parallel genotyping of a large numbers of samples, a streamlined restriction site-associated DNA genotyping method named 2b-RAD (which uses type IIB restriction enzymes) was developed (Wang et al., 2012). Besides, protocols for library preparation were simplified and genome coverage regulation made more flexible (Wang et al., 2012). As this method combines the advantages of low-cost genome-wide genotyping and uniform distribution of markers, 2b-RAD has been utilized in different species for genotyping and association analyses (Seetharam and Stuart, 2013; Pecoraro et al., 2016).

Historically, two approaches have been applied to explore the candidate molecular markers significantly contributing to growth traits: fixation index (*Fst*) estimation based on the genome-wide SNPs information, and quantitative trait loci (QTL). In the past decade, QTL were determined for a number of economically important traits in common carp. For growth traits, Laghari et al. (2014) identified six QTL associated with body weight (BW), body length (BL), and body thickness (BT), and explained phenotypic variance in the range of 17.0–32.1%. In order to determine whether identified QTL can be applied across different common carp strains, Gu et al. identified three QTL for body weight, conserved both in mirror carp and Jian carp strains (Gu et al., 2015). Although conserved QTL were found, the specific pathways regulated by those QTL remained unexplored. Zheng et al. relied on the reference genome data (Zheng et al., 2017) to explore a QTL located in a significantly upregulated actin cytoskeleton pathway on the chromosome LG45 using 250 microsatellite markers. This pathway plays a major role in the development of neurons and in structural changes of adult neurons (Luo, 2002). Although this approach can be used to successfully annotate the candidate QTL, a large number of molecular markers needs to be studied. High-density array method was traditionally used to capture a large number of genome-wide SNPs associated with different QTL (McCue et al., 2012; Houston et al., 2014). For example, in common carp, Zheng et al. detected 18 QTL putatively associated with four different muscle- and body fat-related traits (Zheng et al., 2017). However, high-density array remains too expensive for the practical common carp breeding. Therefore, in this study, we took advantage of our newly-established fast-growing carp strain to attempt to apply the cheaper 2b-RAD method to identify, annotate and functionally verify growth-associated genome-wide SNPs.

The other traditionally used method to explore candidate molecular markers useful for breeding programs is the estimation of fixation index (*Fst*) on the basis of genome-wide SNP information. Al Abri et al. identified a correlation between the locus of *ANKRD1* gene and withers height in horses under a dominant mode of inheritance using genome-wide *Fst* estimation and cross-population composite likelihood ratio test (Al Abri et al., 2017). Yang et al. calculated pairwise *Fst* values between Chinese indigenous and commercial pig breeds and found evidence of positive selection on the *insulin-like growth factor 1 receptor* gene (Yang et al., 2014a). Therefore, *Fst*-based approach can be used to examine the candidate genes for the selective breeding. On the basis of these findings, we designed our study of candidate loci for increased growth performance of the new *Xinlong* carp strain to include a large number of families. As 208 SNPs with significantly elevated *Fst* values were identified previously between strains of sheep susceptible and resistant to ivermectin in *Haemonchus contortus* using 2b-RAD technology (Luo et al., 2017), we hypothesized that cost-effective 2b-RAD technology can be used to explore the SNPs behind the faster growth of the new *Xinlong* strain.

Although both QTL and *Fst* estimation are effective methods for gene function discovery, they also have shortcomings (Korte and Farlow, 2013). Whereas, QTL mapping within full-sibling families provides low resolution, but high statistical power to identify regions of the genome that cosegregate with a given trait either in F2 populations or in Recombinant Inbred Line (RIL) families, *Fst* analysis provides good resolution, but its QTL detection power is influenced by the frequency of alleles and it is sensitive to the population structure. As this can result in false positives, the method requires a lot of verification work. Therefore, the objective of this study was to rely on genome-wide SNPs to combine QTL and *Fst* analyses to explore growth-related functional genes and loci in the new *Xinlong* strain of common carp. To achieve this, we compared the fast-growing new strain with the slow-growing control population of Huanghe carp. This study aims to use this approach not only to help breeders improve the growth performance of cultured fish (and other animal) species, but also attempts to address the much broader question of the genetic control of growth in animals in general.

## Materials and Methods

### Fish

A total of 250 common carp specimens belonging to 25 full-sib families (10 specimens/family: 8 offspring + 2 parents) were selected according to their breeding values predicted using BLUP (Ponzoni et al., 2005) from the Nanquan farm of the Freshwater Fisheries Research Center, Chinese Academy of Fishery Sciences. The population included 19 “selection” families (the newly selected *Xinlong* strain) and 6 “control” families (traditional Huanghe strain). These families were generated by artificially stimulating female brooders for spawning with hormonal injections, and then manually mixing the roe with milt from the matched male brooder. The fertilized eggs were then transferred to labeled hatching hapas (cage settle nets), and a week later larvae were transferred to separate nursery hapas. Each family was assigned a separate hapas, a unique ID, and spawning times were recorded.

Fine-mesh hapas were set up in one row within a concrete tank during both stages (fertilization and nursery). The size of the hapas was 120 cm (length) × 80 cm (width) × 100 cm (depth). Tanks were supplied with filtered well water and equipped with air stones for continuous aeration. Throughout the experiment, the dissolved oxygen ranged from 3.8 to 8.6 mg/L, pH from 7.2 to 8.5, and temperature from 18 to 20°C (avg. = 19.40 ± 0.02). Fish were fed *ad libitum* (daily about 5% of their body weight), with a commercial feed containing 30% of protein. After about 3 months, when they reached about 10 cm in length, eight fish were randomly sampled from the offspring of each family and tagged individually with passive integrated transponder tags (PIT) produced by Biomark. Their body weight and body length (abbreviated as iBwt and iBlen, respectively) were measured.

Five months later, the fish were anesthetized with clove oil (75 mg/L) (Velisek et al., 2005), and body weight, length, width and depth (abbreviated as Bwt8m, Blen8m, Bwd8m, and Bdp8m, respectively) of each individual were recorded again to analyse the growth performance. The fish were then immediately euthanized using 0.2 mg/mL tricaine methanesulfonate (Sigma, USA), dissected, and 50–100 mg samples of caudal fins were collected. The dissected caudal fin tissues were flash frozen in liquid nitrogen, transported to the laboratory, and stored at −70°C until DNA extraction.

Animal handling and experimental procedures were carried out in accordance with the guidelines for the care and use of animals for scientific purposes set by the Institutional Animal Care and Use Committee of the Freshwater Fisheries Research Center, and approved by the animal ethics committee of Chinese Academy of Fishery Sciences.

### 2b-RAD Library Preparation and Sequencing

Genomic DNA was isolated from the caudal fins using the Universal Genomic DNA Extraction Kit Ver. 3.0 (Takara, Japan) according to the manufacturer's instructions. Concentration and quality of the DNA were verified by spectrophotometry (optical density reading at 260 and 280 nm) and electrophoresis on 1.0% agarose gel.

2b-RAD libraries were constructed following adaptor and primer sequences, as well as the protocol, reported by Wang et al. (2012): 500 ng of DNA per specimen was digested in a 6 mL reaction volume using 1 U BsaXI at 37°C for 1 h, followed by enzyme heat inactivation at 65°C for 20 min. These digestion products were linked with T4 DNA ligase at 16°C for 3 h, with subsequent heat inactivation for 10 min at 65°C. PCR products were purified using the SPRIselect purification kit (Beckman Coulter, Pasadena, CA, USA) and quantified through a Qubit 2.0 Fluorometer (Invitrogen). The quality of all amplicon libraries was checked on 1.8% agarose gels and verified on Agilent 2100 Bioanalyzer. Finally, pooled libraries were sequenced on an Illumina HiSeqXten platform (Illumina, San Diego, CA, USA) using 150 base pair-end sequencing. All of the 2b-RAD sequences were archived in the NCBI SRA database (SRR6241620 and SRR6262716).

### Quality Filtering and Genotyping

Quality check (QC) of sequenced reads and adapter trimming were performed by running a customized script (Pecoraro et al., 2016), to obtain 27 bp-long fragments. SNP calling was performed using STACKS v1.23 (Catchen et al., 2013). For each family, individual genotypes were constructed using components of the STACKS pipeline as follows: (i) Ustacks program was employed for building loci from all QC-passed reads using the following parameters: m3, M2, and N4; (ii) Genotyping was performed using procedures described by Jiao et al (Jiao et al., 2014). Terminal 3-bp positions were excluded from each read to eliminate artifacts that might have arisen at ligation sites. Reads with no restriction sites or containing ambiguous base calls (N), long homopolymer regions (>10 bp), excessive numbers of low quality positions (>5 positions with quality of < 10), were removed. The remaining trimmed, high-quality reads formed the basis for subsequent analyses (Jiao et al., 2014).

### Candidate SNP Detection and Gene Annotation

The phenotypic value is decomposed as:

(1) $$\begin{array}{r} y=Xb+Sc+Zg+Wq+e \end{array}$$

where ***y*** is the vector of observation value; ***b*** is the vector of fixed effect including the population mean and selection line effect; ***c*** is the vector of cage effect, and ***c***~*N*(0, **I**$\sigma_{c}^{2}$), $\sigma_{c}^{2}$ is the variance of the cage component; ***g*** is the vector of additive genetic effect explained by polygenes, ***g***~*N*(0, **K**$\sigma_{g}^{2}$), where **K** is realized genetic relationship matrix calculated from genome-wide SNP information (VanRaden, 2008) and $\sigma_{g}^{2}$ is the genetic variance explained by polygenes; ***q*** the is vector of allele substitution effects of the major QTL, which are treated as fixed effects; ***X***, ***S***, ***Z***, ***W*** are the corresponding design matrices for ***b***, ***c***, ***g*** and ***q***. ***e*** is the vector of residuals: ***e***~*N*(0, **I**$\sigma_{e}^{2}$), where $\sigma_{e}^{2}$ is the variance of random error. The overall phenotypic variance-covariance can be expressed as:

$$V=\sigma_{c}{}^{2}SS\prime +\sigma_{G}^{2}ZKZ\prime +\sigma_{e}{}^{2}I$$

The mixed model equations for the model (1) are:

$$\left[\begin{array}{cccc} X^{{}^{\prime }}X & X^{{}^{\prime }}W & X^{{}^{\prime }}S & X^{{}^{\prime }}Z\\ W^{{}^{\prime }}X & W^{{}^{\prime }}W & W^{{}^{\prime }}S & W^{{}^{\prime }}Z\\ S^{{}^{\prime }}X & S^{{}^{\prime }}W & S^{{}^{\prime }}S+\lambda_{1}I & S^{{}^{\prime }}Z\\ Z^{{}^{\prime }}X & Z^{{}^{\prime }}W & Z^{{}^{\prime }}S & Z^{{}^{\prime }}Z+\lambda_{2}+K^{-1} \end{array}\right]\left[\begin{array}{c} \overset{\wedge }{b}\\ \overset{\wedge }{q}\\ \overset{\wedge }{c}\\ \overset{\wedge }{g} \end{array}\right]=\left[\begin{array}{c} X^{{}^{\prime }}y\\ W^{{}^{\prime }}y\\ S^{{}^{\prime }}y\\ Z^{{}^{\prime }}y \end{array}\right]$$

Where $\lambda_{1}=\sigma_{e}^{2}/\sigma_{c}^{2}$ and $\lambda_{2}=\sigma_{e}^{2}/\sigma_{g}^{2}$. The estimated genomic breeding values are expressed as: GEBV = Zg + Wq, and heritability of a trait is: $h^{2}=\left(\sigma_{q}^{2}+\sigma_{g}^{2}\right)/\left(\sigma_{q}^{2}+\sigma_{g}^{2}+\sigma_{c}^{2}+\sigma_{e}^{2}\right)$q, where $\sigma q_{{}^{2}}$ is the variance of the breeding effect explained by all major QTL. Association analysis was performed using the StepLMM method (Lin et al., 2017).

Differentiation index *Fst* between selection and control groups was calculated using the VCF tool v0.1.11 (Danecek et al., 2011). SNPs with strong selection signature were examined by differential *Fst* values on genome-wide information (threshold value was 1% *Fst*). Manhattan graph of genome-wide *Fst* with window size 100,000 was plotted using Python2.7. The principal component analysis (PCA) was conducted by plink 1.07 (Purcell et al., 2007) and GCTA 1.13 (Yang et al., 2011). We identified candidate growth-associated genes or loci using the following criteria: (i) the genes contained the above-mentioned SNP loci with signature of selection; (ii) the genes were annotated. Functional information and annotation of genes were conducted by inquiring the common carp reference genome (Xu et al., 2014b).

### Generation of Mutant *IGF2a* and *IGF2b* Zebrafish

As the QTL analysis identified two adjacent *IGF2* genes (*IGF2a* and *IGF2b*) as the foremost candidate locus for different growth performance of the two strains, to further explore functions of these two genes in fish, we used CRISPR–Cas9 strategy to generate IGF2a- and IGF2b-knockout mutant zebrafish lines. Two guide RNAs (gRNAs) were designed, targeting exons of zebrafish *IGF2a* and *IGF2b* (Supplementary Table S1). gRNAs were cloned into the pT7gRNA vector as described (Lin et al., 2016) (oligonucleotide sequences are given in the Supplementary Table S1). gRNAs were transcribed using the MEGAscript T7 Transcription Kit and purified using the mirVana miRNA isolation kit. Cas9 mRNA was synthesized using the mMessage mMachine Sp6 Transcription Kit (all three kits were from Thermo Fischer Scientific, AM1334, AM1560 and AM1340 respectively) and purified using the RNA cleanup protocol from the RNAeasy mini kit (Qiagen 74104).

Wild-type AB strain zebrafish were injected at the one-cell stage with ~50 ng gRNA and ~100 ng Cas9 RNA. These F0 fish (*n* = 100) were raised to maturity and genotyped using fin clipping, DNA isolation and PCR spanning the target site (genotyping primers are given in the Supplementary Table S1). PCR products were analyzed for mutations by Sanger sequencing. Mutant PCR products were cloned into the pMD18-T vector (Takara, Dalian, China) and sequenced to identify the fish carrying the target frameshift mutation. These carrier fish were then back-crossed again to the AB wild type and the resulting F1 fish (*n* = 80) were raised to maturity. The F1 fish were genotyped (same as F0) to identify heterozygous mutant fish followed by cloning and sequencing of the PCR products to validate the presence of the frameshift allele.

## Results

### Growth Performance

**Figure 1:**
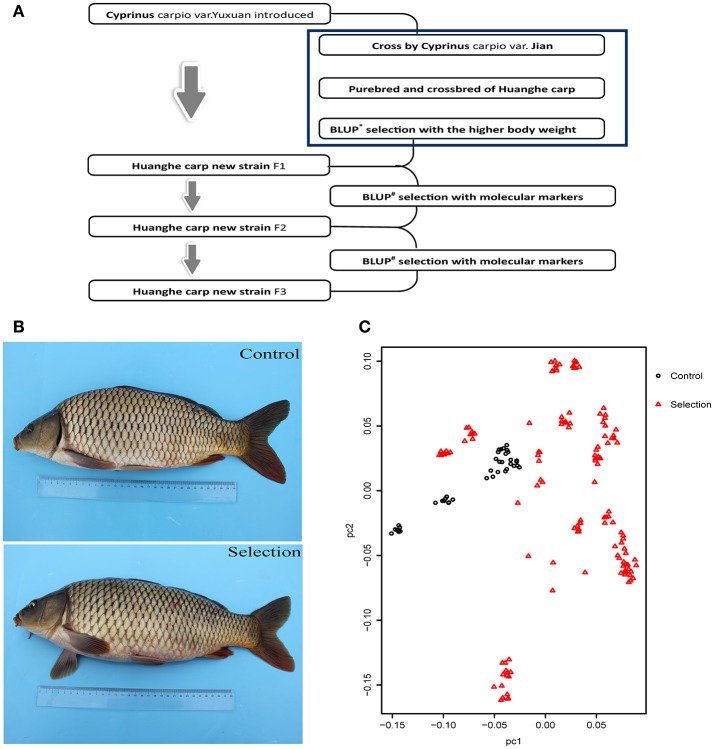
Generation and growth of the new Huanghe carp strain (*Xinlong*). **(A)** *Xinlong* strain selection progress overview. **(B)** Traditional Huanghe carp strain (Control) and the new *Xinlong* strain (Selection). **(C)** Principal Component analysis: the first dimension PC1 was assigned to X axis and the second dimension PC2 was assigned to Y axis. Red triangles represent the selection group, black circles the control group.

The creation of the *Xinlong* strain (Figures 1A,B) was officially recognized after 4 generations of selection (Su et al., 2018). Except for the body length gain, which was non-significantly higher in the control group, all measured growth performance indices were significantly higher (*P* < 0.05) in the selected group than in the control group (Table 1).

**Table 1:** Growth performance of the selection line and control line.

**Trait**	**Selection line**	**Control line**
iBwt^*^ (g)	27.04 ± 0.96^a^	17.47 ± 1.66^b^
Bwt8m^†^ (g)	46.72 ± 1.10^a^	43.85 ± 2.04^b^
Body weight gain (g)	21.95 ± 1.10^a^	17.06 ± 1.98^b^
iBlen^§^ (mm)	100.31 ± 1.32^a^	83.88 ± 2.27^b^
Blen8m^||^ (mm)	120.89 ± 1.56^a^	106.16 ± 2.80^b^
Body length gain^¶^ (mm)	19.78 ± 1.12	21.68 ± 2.01
Bdp8m (mm)	22.02 ± 0.51^a^	18.55 ± 0.48^b^
Bwd8m (mm)	39.81 ± 0.84^a^	34.97 ± 0.67^b^

* *Body weight at tagging*;

† *Body weight 5 months after the tagging; Bwt8m **–** iBwt*;

§ *Body length at tagging*;

|| *body length 5 months after the tagging*;

¶ *Blen8m **–** iBlen. Different character represent the significant difference*.

### Genotypes

2b-RAD libraries were sequenced for 250 specimens (190 selected and 60 control), generating a total of 4,953,017,786 clean reads with a BsaXI Restriction Enzyme site after filtering. Average number of reads per sample was 19,812,071 (Supplementary Table S1). Finally, 56,023,795 unique tags (on average 224,095 per specimen) with average sequencing depth of 43.36 were obtained. Mapping successfulness rates of these tags ranged from 46.76 to 51.11%. A total of 56,663 SNPs were found, covering 50 chromosomes and 3,377 scaffolds (Supplementary Table S2). SNPs were mostly located in intergenic regions (45.07%) and introns (37%) (Supplementary Figure 1; Supplementary Table S3).

### Genetic Differentiation

**Figure 2:**
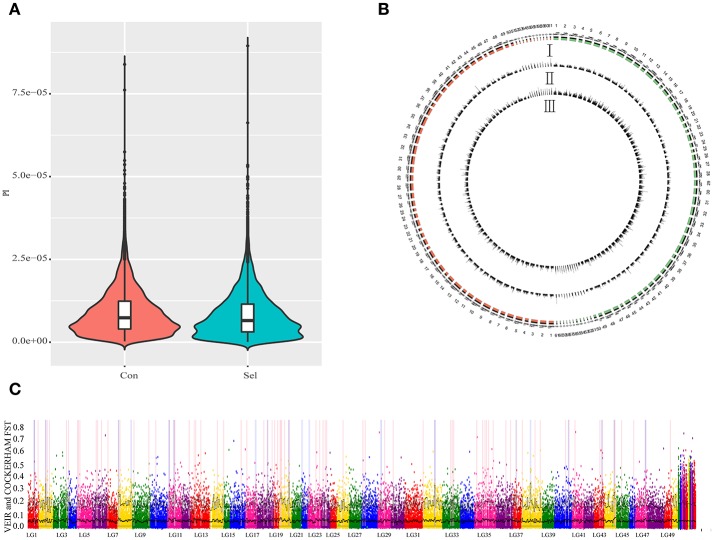
Genome-wide genetic diversity (π) between control and selection groups. Con: control group; Sel: selection group. **(A)**. A violin plot of genetic diversity π for control and selection groups for all chromosomes. **(B)** Genome-wide patterns of nucleotide diversity π for the selection group and control group in G3 offspring in a 2 M window; I represents chromosomes in both selection group (red) and control group; II represents genetic diversity (π) in a corresponding chromosome; III represents the number of SNPs observed per window. **(C)** Gray dots show pairwise *Fst* estimates for single SNPs and black lines show *Fst* means (bold) and 95% quantiles in 1 Mb-wide, non-overlapping windows across the genome. Windows with elevated differentiation are highlighted with blue background frames (mean *Fst* > 0.05) and red background bars (95% quantile *Fst* > 0.25).

To examine the genetic structure of these two groups, we conducted principal component analysis (PCA) based on genomic SNPs. Selection line showed more variation in PC1 and PC2 scores than the control line (Figure 1C). To test whether selection and control lines exhibit marks of artificial selection pressures, genome-wide genetic diversity was measured by nucleotide diversity π (Figures 2A,B). Both lines had similar average genetic diversity: selection π = 8.15 × 10^−6^, and control π = 9.00 × 10^−6^ (Figure 2A). Interquartile ranges (a measure of statistical dispersion, being equal to the difference between 75 and 25th percentiles) were 3.06 × 10^−6^-1.15 × 10^−5^ in selection line and 3.95 × 10^−6^-1.24 × 10^−5^ in control line. All distributions were right-skewed, with asymmetry coefficients ranging between 1.49 (selection line) and 1.53 (control line). Frequency distribution of π values within non-overlapping 2 Mb windows across the genome was similar in both lines (Figure 2B). In order to find out whether the genes involved in the most variable region in the selected strain are beneficial for further selection for target traits, we defined 1,158 domains with the top 50 π values in the selection line as highly variable genomic regions. Annotation revealed that genes found in these highly variable genomic regions belonged to several functional classes: fatty acid metabolism [17 beta-hydroxysteroid dehydrogenase type 8, *HSD17B8* (Chen et al., 2009), receptor expression enhancing protein2, *REEP2* (Ilegems et al., 2010; Hurt et al., 2014); nervous regulation system neuromuscular junction gene Ligand of Numb, protein X 1, *Lnx1*, Young et al., 2005]; Neuronal development *REEP2* (Ilegems et al., 2010; Hurt et al., 2014); sex-related genes [*HSD17B8*, involved in conversion of estradiol to estrone (Villar et al., 2007)]; and NF-κB activation regulation genes [Regulator of G-protein signaling, *EGL-10* and pleckstrin domain containing 7 (*DEPDC7*), (D'Andrea et al., 2014)].

To detect the locus of artificial selection associated with the growth performance of the selected *Xinlong* strain, we scanned all chromosomes with a non-overlapping 2 Mb window and calculated the *Fst* for each window (Figure 2C). Top 1% of *Fst* values were identified as artificial selection loci (in total 244; Supplementary Table S4). Among them, 50.41% were located in intergenic regions, followed by intronic regions (37.7%) (Supplementary Figure 2). Further analyses were focused only on those loci which could be associated with specific genes. Some of them were associated with sex factors, such as 17β-Hydroxysteroid dehydrogenase 3 (*HSD17B3*), which is regarded as a male reproductive marker (Hoffmann et al., 2016), and SPARC-related modular calcium binding 2 (*SMOC2*), involved in gonad and reproductive tract differentiation (Pazin and Albrecht, 2009; Roy et al., 2013). Other were mostly related to nutrition metabolism: Ras homolog enriched in brain (*RHEB*), involved in skeletal myogenesis through suppression of insulin and hypothalamic neurons that regulate food intake and peripheral metabolism (Ge et al., 2011; Yang et al., 2014b; Meng et al., 2017); Ras-related protein Rab-10 (*RAB10*), related with insulin-stimulated glucose uptake in adipocytes and spermiogenesis (Karunanithi et al., 2014; Sano et al., 2015; Lin et al., 2017); vasoactive intestinal peptide receptor 2 (*VIPR2*), associated with muscle mass and force, lipolytic effects, obesity, oocyte maturation and circadian rhythms (Akesson et al., 2005; Hinkle et al., 2005; Liu et al., 2010; Zhou et al., 2011; An et al., 2012). Among the metabolism-related genes, a number of genes could be directly related to the lipid metabolism: acyl-CoA synthetase family member 2 (*ACSF2*), related to long-chain fatty acid metabolic processes (Pashaj et al., 2013); tenomodulin (*Tnmd*), related to adipocyte differentiation and beneficial visceral adipose tissue expansion (Senol-Cosar et al., 2016); and Syntrophin beta 1 (*SNTB1*), related to HDL cholesterol metabolism (Okuhira et al., 2005). Finally, Tran membrane channel like 7 (*TMC7*) and Collagen α-1 (XXIII) chain (*COL23A1*) were associated with milk production traits in Holstein cows (Lee et al., 2016b); and transient receptor potential cation channel subfamily M member 7 (*TRPM7*) was associated with osteogenic induction (Liu et al., 2015).

### QTL Analysis

**Figure 3:**
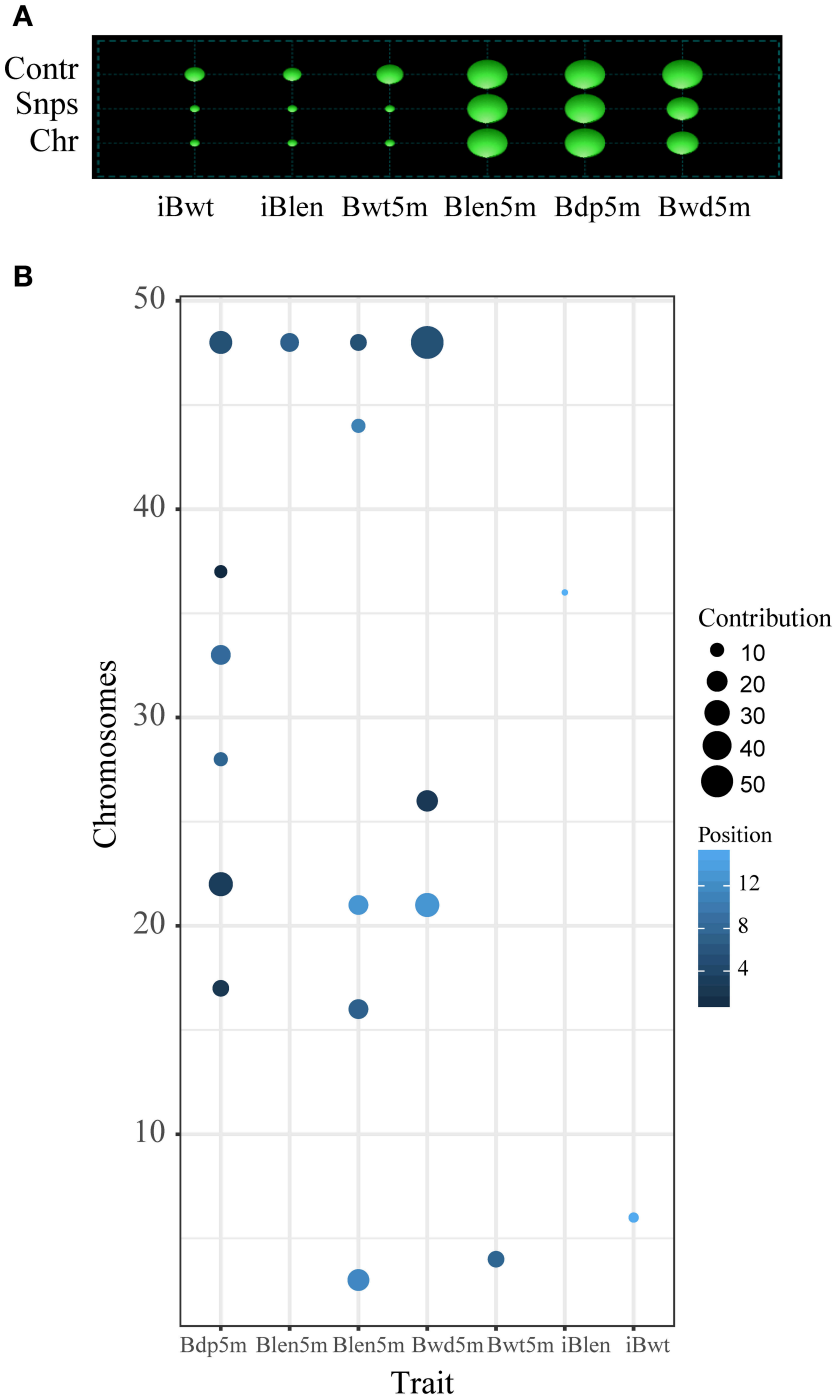
QTL analysis for the *Xinlong* strain based on genome-wide SNPs. Chr: the number of chromosomes which were associated with target traits; Snps: the number of SNPs associated with a specific trait; Contr: contribution of SNPs to specific traits. Size of cycle corresponds to the value magnitude. **(A)** Relationship between specific traits (horizontal axis) and their corresponding total number of SNPs and chromosomes (vertical axis). **(B)** Relationship between specific traits (horizontal axis) and their corresponding SNPs and chromosomes.

In order to study the differences between the results of *Fst* and QTL analyses, the QTL analysis was conducted as described before (Li et al., 2017): a total of 18 QTL, located on 14 chromosomes, were identified for the 6 studied growth traits (Tables 1, 2). Two overlapping QTL for Bwd8m and Blen8m were located on chromosomes 21 and 48. One of these two was also associated with Bdp8m. More than two QTL were associated with Blen8m, Bwd8m, and Bdp8m, whereas iBwt, iBlen, and Bwd8m had only one QTL associated with them (Figure 3). Some growth traits were associated with a larger number of QTL with smaller individual contributions, whereas others were under the control of a smaller number of QTL with higher individual contributions (Figure 3A). For example, Bdp8m was associated with a smaller number of SNPs and high contribution from individual chromosomes (Figure 3A). Among all studied traits, Bwd8m exhibited the highest contribution from a single QTL (Figure 3B). In the regions where identified QTL were located, we found three classes of functional genes: nervous system-associated genes, signal regulation pathways-associated genes, and metabolism-associated genes (Supplementary Table S5).

**Table 2:** QTL associated with growth performance of the *Xinlong* strain.

**Trait**	**Chromosome**	**Position**	Cp^**^	**Accession number**
iBwt	6	15084622	0.07	NC_031702.1
iBlen	36	15736824	0.06	NC_031732.1
Bwt8m	4	7699816	0.13	NC_031700.1
Blen8m	48	7056033	0.16	NC_031744.1
Blen8m	3	11692346	0.22	NC_031699.1
Blen8m	16	7248359	0.18	NC_031712.1
Blen8m	21	12933756	0.18	NC_031717.1
Blen8m	48	5203500	0.13	NC_031744.1
Blen8m	44	10794140	0.10	NC_031740.1
Bdp8m	48	5203500	0.24	NC_031744.1
Bdp8m	17	2113489	0.13	NC_031713.1
Bdp8m	33	8572666	0.18	NC_031729.1
Bdp8m	22	2786001	0.27	NC_031718.1
Bdp8m	37	479269	0.09	NC_031733.1
Bdp8m	28	7679394	0.10	NC_031724.1
Bwd8m	21	12933756	0.27	NC_031717.1
Bwd8m	26	2443963	0.21	NC_031722.1
Bwd8m	48	5203500	0.52	NC_031744.1

** *Contribution to the phenotype variance*.

Heritability values of the six growth indices were medium or high, ranging from 0.36 for Bwt8m to 0.59 for Blen8m (Table 3). StepLMM analysis was used to analyze the genetic variance components. The results showed that genetic variance of Bwd8m and Bdp8m and Blen8m can be attributed mostly to the effects of QTL (Table 3). Bwt8m and iBlen exhibited the highest polygenic effects, whereas Blen8m had the highest genetic, error and QTL variances. Importantly for breeding programs, Bdp8m and Blen8m exhibit a combination of most of their genetic variance accounted for by QTL and high heritability.

**Table 3:** Estimated heritability of growth traits in the *Xinlong* strain.

**Trait**	**Vp^††^**	**Vg^‡‡^**	**Ve^§§^**	**Vq^¶¶^**	**Heritability**	**Vq/Vg**
iBwt	79.02	62.51	0.19	5.62	0.48	0.07
iBlen	121.84	81.79	13.98	7.66	0.42	0.06
Bwt8m	137.48	77.37	84.30	19.87	0.36	0.13
Blen8m	14.02	519.54	368.94	520.77	0.59	0.97
Bdp8m	0	15.57	11.68	15.58	0.57	1
Bwd8m	0	26.19	44.28	26.19	0.37	1

†† *Polygenic effect variance; Genetic variance*;

§§ *Error variance*;

¶¶ *QTL variance*.

### Combining *Fst* and QTL Analyses

**Figure 4:**
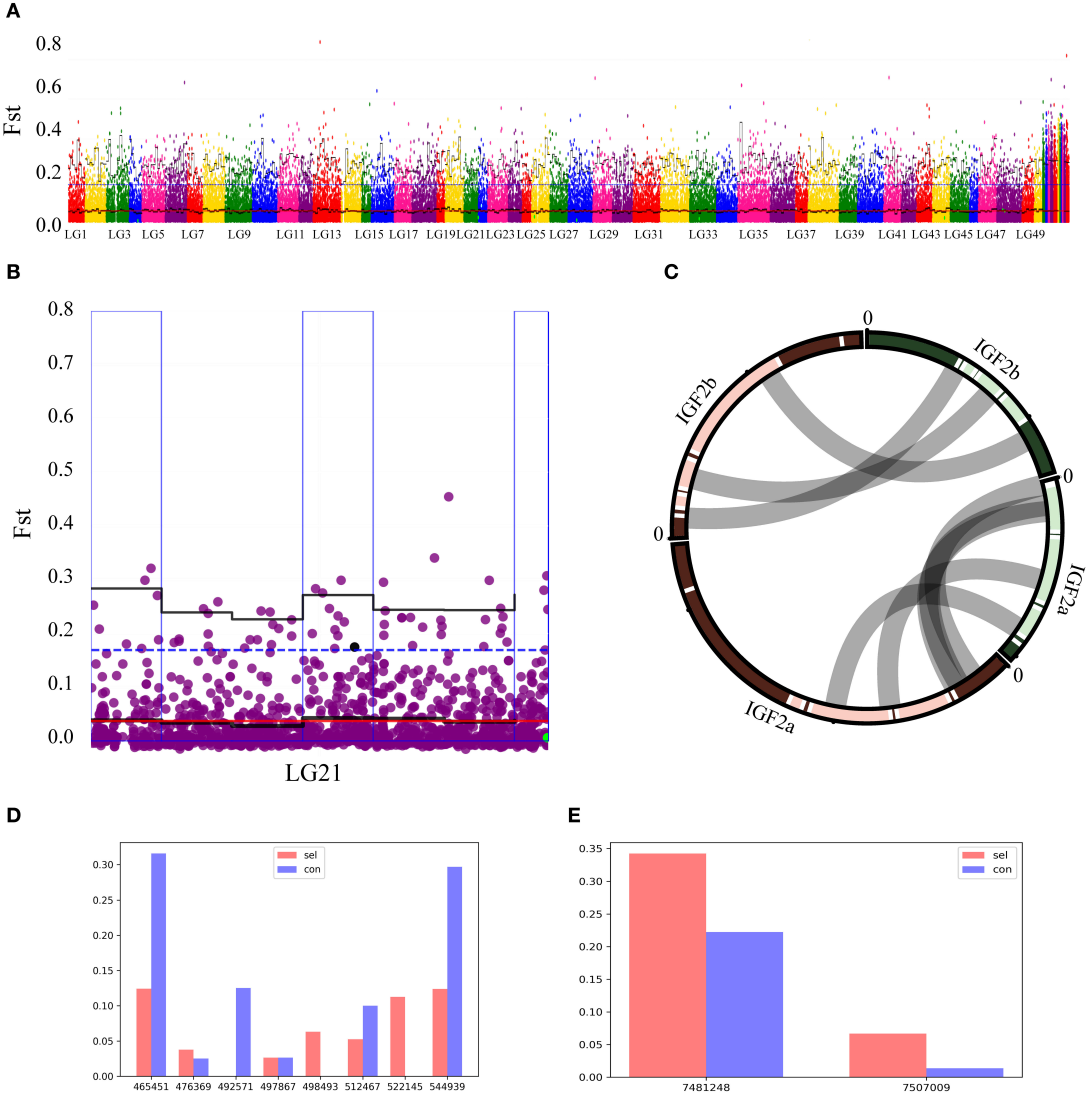
Overlap between *Fst* and QTL analyses. **(A)** Gray dots show pairwise *Fst* estimates for a single SNP, and black lines show *Fst* means (bold) and 95% quantiles in 2 Mb wide, non-overlapping windows across the genome. Blue dotted line represents top 5% of the *Fst* values. Red line represents the average *Fst* value. Windows with elevated differentiation are highlighted with blue background frames (mean *Fst* > 0.05) and red background bars (95% quantile *Fst* > 0.25). **(B)** Overlap between the *Fst* (blue outer frame box) and QTL on the chromosome 21 in the region where *IGF2b* was found. Black line represents the average *Fst* value in a 2M window, blue dotted line represents *Fst* = 0.5 value. **(C)** *IGF2a* and *IGF2b* sequence alignment between common carp and zebrafish demonstrate highly conserved residues for these two species. Their positions on chromosomes are linked by gray lines. **(D,E)** Genome-wide SNPs of *IGF2a* and *IGF2b* observed in control (Con) and selection (Sel) groups. **A** *IGF2a*; b; *IGF2b*; horizontal axis represents all SNPs in the ± 5 kb region (upstream 5 kb to downstream 5 kb) around these two genes; vertical axis respectively the frequency of a locus.

QTL explored above were appended to the Manhattan map of *Fst* values (Figure 4A). After filtering the overlapping regions, where observed QTL were located in 95% quartile boxes within 1Mb windows, one box on the chromosome 21 was identified as a candidate locus for growth performance (Figure 4B). Two *IGF2* sequences (*IGF2a* and *IGF2b*) were annotated in this region of the common carp genome (Xu et al., 2014b). As most mammals and teleost fishes possess only one *IGF2* gene copy, in order to assess the (dis)similarity of these two sequences, Su et al. cloned *IGF2a* and aligned it with *IGF2b*, and proposed homology between these paralogs in common carp and zebrafish (Su et al., 2012). We aligned the two genomic regions containing these two genes, including the upstream and downstream sequences, and found a high similarity of the two paralogs to their zebrafish orthologs (Figure 4C). Frequencies of all SNPs in the vicinity of these two genes (upstream 5 kb and downstream 5 kb) were plotted (Figures 4D,E). In the *IGF2a* sequence, only A was found on the position 492,571 in the selection group, whereas only T was found on the positions 498,493 and 522,145 in the control group (Figure 4D). In the *IGF2b* sequence, G frequency on the position 7,481,248 and T frequency on the position 7,507,009 were higher in the selection group than in the control group (Figure 4E). These results suggest that *IGF2a* and *IGF2b* genes might be associated with the growth rate of this fish.

### *IGF2a* and *IGF2b* Knockout in Zebrafish

**Figure 5:**
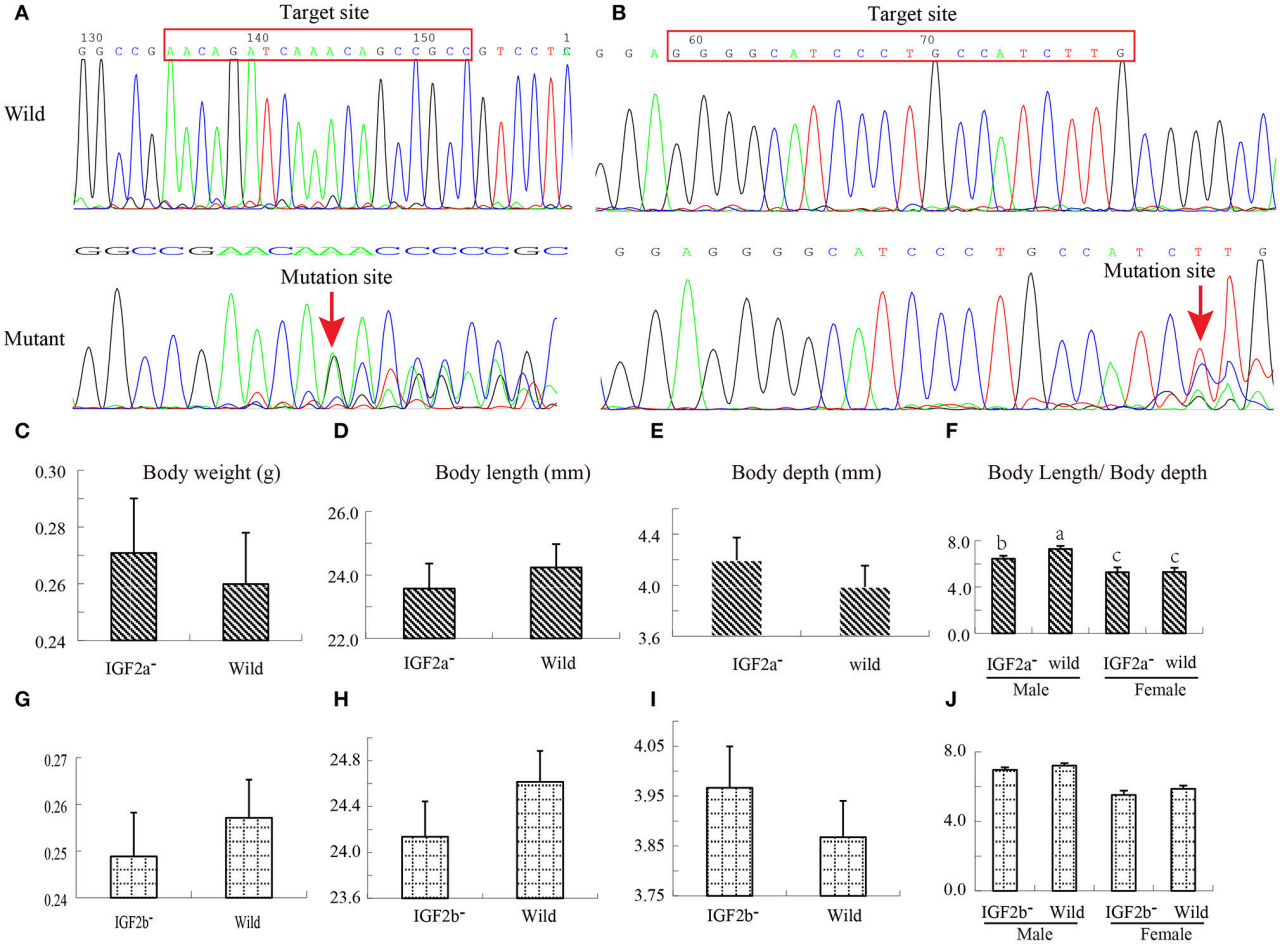
Construction of *IGF2a* and *IGF2b* knockout zebrafish and their growth performance. **(A)** Target loci and Sanger sequencing results showing that *IGF2a* gene has a frameshift mutation. **(B)** Target loci and Sanger sequencing results showing that *IGF2b* gene has a frameshift mutation. **(C–F)** Differences in body weight, body length, body depth and body length/body depth ratio between wild and *IGF2a*-knockout zebrafish. **(G–J)** Differences in body weight, body length, body depth and body length/body depth ratio between wild and *IGF2b*-knockout zebrafish.

To test this hypothesis, we used CRISPR–Cas9 to generate IGF2a-knockout and IGF2b-knockout mutant zebrafish lines. We confirmed the successful knockout by sequencing these genes in wild and mutant types; sequence comparison shows a frameshift mutation (Figures 5A,B). Both mutant lines exhibited differences (albeit mostly above the statistical significance threshold) in growth traits in comparison to the wild type (Figures 5C–J). The IGF2a-knockout fish were shorter but heavier and deeper-bodied than the wild type (Figures 5C–F), whereas the IGF2b-knockout fish were also shorter and deeper-bodied, but lighter than the wild type (Figures 5G–J). Intriguingly, for *IGF2a* these differences in body weight/length ratio were more strongly pronounced in males (the only statistically significant difference; Figures 5F,J). These results indicate that both genes might be involved in growth regulation in zebrafish, but further studies are needed to corroborate this. We hypothesize that nucleotide substitutions might regulate the growth by influencing the transcription factor binding (Laurila and LãHdesmãKi, 2009) or by influencing the protein structure (Perng et al., 1999).

## Discussion

Selection on a limited number of markers for quantitative traits often misses a substantial portion of the genetic variance contributed by loci with small effects (Poland et al., 2012). As a result of this, although genomic breeding facilitates direct selection for favorable alleles in selective breeding schemes, it is not considered to be cost-effective due to a large number of genome-wide markers required for its successful application. Usually, high-density or low-density assays were used in animal breeding, with such examples as Bovine SNP50 chip with 54,001 SNP loci to support genome wide association applications in cattle (Matukumalli et al., 2009), and BovineLD chip with 6,909 SNPs, which aimed to facilitate low-cost GS for taurine in beef and dairy cattle (Boichard et al., 2012). A combination of the two (high- and low-density assays) was proposed as a promising strategy for the implementation of GS at acceptable costs, where a panel size of 384 markers could be recommended for the selection of candidates for pig breeding programs if at least one parent of selection candidates is genotyped at high-density (Wellmann et al., 2013). In common carp, although a high-density array with 250,000 genome-based SNPs had been developed (Xu et al., 2014a), this array was only used to detect the QTL for growth and sex-related traits (Peng et al., 2016). However, a cost-effective combination of high-density SNP genotyping platform and capturing genome-wide SNPs for a large number of specimens remains unavailable in practice. In order to explore the candidate molecular markers for GS in the *Xinlong* strain, we mapped the total of 56,663 SNPs on the genome-wide level for parents and their offspring using the 2b-RAD methodology. To ensure that our results have broad applicability, specimens from 25 different families were selected to form the selection and control groups. Genetic diversity analysis indicates that selection line was as genetically diverse as the heterogeneous sample set of the control line. Thus, selection did not cause a major genetic diversity loss in the *Xinlong* strain, and we can continue the breeding program for this population.

### Features of QTL and *Fst* Methods in Exploring the Candidate Loci

As previous QTL analyses in common carp relied on data from only one family (Laghari et al., 2014; Lu et al., 2015), it remains unclear whether these QTL are applicable to multifamily studies, or whether this approach identified only a small proportion of useful QTL due to high genetic relatedness of the sample. Moreover, as Lv et al. found that growth-related (BW, TL, and BT) QTL only partially overlapped between families in their multifamily study, this indicates that a single-family approach does not necessarily produce results applicable across different families (Lv et al., 2016). Therefore, a multifamily approach is needed to study growth-related QTL in the *Xinlong* strain. In comparison to QTL, an advantage of the fixation index estimation is that it can also examine the candidate genetic regions with selective signals. Flori et al. identified 13 regions subjected to strong and/or recent positive selection in cows by estimating *Fst* values, and found that some of them contained genes with a strong effect on milk production (Flori et al., 2009). Moon et al. reported that genes with strongest signals of directional selection for traits important for pig reproduction and production under artificial selection belong to the group III of metabotropic glutamate receptors (Moon et al., 2015). Similarly, candidate SNPs should be tested for their across-population applicability. Zhao et al. applied two complementary methods to detect selection signatures in seven different cow breeds: *Fst* was used to detect 704 individual SNPs, and results then compared to the integrated haplotype score (iHS, a measure of the amount of extended haplotype homozygosity at a given SNP along the ancestral allele relative to the derived allele) (Zhao et al., 2015). This indicates that *Fst* can be used to determine the candidate SNPs, or genes, or signals of selection, between selection and control groups in the *Xinlong* strain breeding program.

In summary, the QTL method is sensitive to population structure and has lower resolution, while the *Fst* method has higher resolution. Using the traditional (non-high-throughput) methods of molecular marker data collection (such as microsatellites and restriction fragment length polymorphisms), QTL is a suitable method for target species without a published reference genome. In order to avoid identifying the candidate QTL specific to only one family, we used 25 families and the cost-effective 2b-RAD genotyping capture method to examine candidate molecular markers in the Xinlong breeding population. For this, we modified a method recently developed to estimate the QTL of a limited number of individuals belonging to 8 families (Li et al., 2017). This way, the identified QTL should be applicable to other studies of this strain. In order to further increase the resolution, *Fst* analysis was also used to study candidate markers inferred from genome-wide SNPs. In order to avoid false positive errors, gene knockout technology was used to verify the candidate genes identified by overlapping the QTL and *Fst* approaches.

### QTL and Candidate Genes

#### QTL Discovered

Using 25 full-sib families of the *Xinlong* strain and high throughput sequencing, we identified 18 QTL associated with growth traits, which contributed to genetic variance from 7 to 100%. One QTL located on the chromosome 48 was shared among three growth indices (Blen8m, Bdp8m, and Bwd8m), which may partially explain the interconnectedness of these growth traits in common carp (Su et al., 2013). Although there exists a strong correlation in growth curve dynamics between different growth and developmental stages (Ibáñez-Escriche and Blasco, 2011), which indicates that growth traits in different developmental stages should be controlled by the same QTL (Bartholomé et al., 2013; Würschum et al., 2014), we could not identify any body weight-associated QTL shared between different growth stages. This illustrates that further studies are needed in this aspect. Peng et al. identified 22 QTL for growth-related traits at 18 months of age on the basis of chromosome-wide log odds score (LOD; with significance threshold set at *P* < 0.01), and found that they can explain 15.5–45.1% of the total phenotypic variation (Peng et al., 2016). Compared to our research, they merely reported candidate QTL regions and some genes in these regions, but they did not study SNP positions and their contribution to the phenotypic variance. Beside this, we also determined whether a specific growth trait is controlled by a major QTL effect or a polygenic effect using the StepLMM approach.

#### Neural Regulation may be a Major Component Behind the Higher Growth Performance of the Selection Line

To our knowledge, definitive genes or loci that can explain the growth performance variance mostly remain unavailable for carp breeding programs. Using gene chip information, Peng et al. identified candidate regions containing many important growth performance regulators, such as *KISS2, IGF1, SMTLB, NPFFR1*, and *CPE* (Peng et al., 2016). In our study, several candidate genes for QTL related to growth performance of the *Xinlong* strain were determined, such as *SHANK2* and *MDGA2* for iBwt and Bwt8m, respectively. The former gene, *SHANK2*, was associated with both excitatory and inhibitory synapses (Vaccarino et al., 2009), whereas a mutation in *MDGA2* was shown to elevate excitatory transmission, implying that *MDGA2* blocks neuroligin-1 interaction with neurexins and suppresses excitatory synapse development (Connor et al., 2016). A direct connection between body weight and neural regulation was reported in recent years: multiple neural circuits which can affect the body weight by regulating the balance between food intake and energy expenditure were identified in the brain (Rui, 2013; Rathjen et al., 2017). Two loci were identified near the *cell adhesion molecule 1* and *2* genes (they encode membrane proteins that mediate synaptic assembly); these two loci influenced the expression of these two genes, thereby disrupting energy balance and promoting weight gain (Rathjen et al., 2017). As regards brain-derived neurotrophic factor-like (*BDNF*), which is important for synaptic plasticity, learning and memory production, the serum area under the curve of BDNF significantly decreased after body weight reduction for obese men (Lee et al., 2016a), whereas a reduction in body weight and improved glucose tolerance in obese mice were accompanied by an increase in BDNF levels (Wang et al., 2017).

In common carp, sex has a strong impact on growth performance, as females generally have higher body weight than males. In the present study, a gene named *NRXN1A* was identified as a QTL related to Blen8m. In zebrafish, this gene is expressed in the adult testis and in the earliest stages of development, before the beginning of zygotic transcription (Rissone et al., 2007). Therefore, sex-specific regulation may be involved in the higher Blen8m in the selection line.

#### Candidate Loci Identified by both QTL and *Fst* Analyses

The combination of genome-wide selective signatures and QTL information revealed that genetic differences between the *Xinlong* strain and the control line could be roughly classified into three categories of pathways. (1) Neural pathways: *RHEB* (Yang et al., 2014b; Meng et al., 2017) and *BDNF* are involved in nutritional regulation, including food intake and peripheral metabolism (Meng et al., 2017). *MDGA2, NRXN1a, SHANK2* and *REEP2* genes are involved in physical excitatory transmission process (Vaccarino et al., 2009), while *LNX1* is involved in neuromuscular junction (Vaccarino et al., 2009). Finally, vasoactive intestinal peptide receptor 2 (*VPAC2*) regulates the muscle mass and force (Ilegems et al., 2010; Hurt et al., 2014), which is directly related to growth performance. (2) Sex pathways. Genes implicated in these pathways can be divided into two main subcategories: gonad development and sex hormone regulation of growth stimulation. Three genes are associated with the former subcategory: *NRXN1A, SMOC2*, and *RAB10* are involved in gonadogenesis, specifically spermiogenesis and testes development (Young et al., 2005). Two genes from the same family are associated with the latter category: *HSD17B3* is a male reproductive marker (Pazin and Albrecht, 2009) which can take part in the production of the male sex hormone, testosterone, from a precursor hormone called androstenedione, and *HSD17B8* is involved in the conversion of estradiol (E2) to estrone (E1) (Lin et al., 2016). (3) Fatty acid metabolism regulation pathways: *RAB10* is involved in insulin-stimulated glucose uptake in adipocytes (Lee et al., 2016b; Senol-Cosar et al., 2016). Tenomodulin (*TNMD*) is associated with adipocyte differentiation and beneficial visceral adipose tissue expansion (Villar et al., 2007). As opposed to the above fat deposition-related genes, *VPAC2* has lipolytic effects (Karunanithi et al., 2014); which suggests that the faster growth of the *Xinlong* strain is a consequence of a complex bidirectional balance of fat metabolism regulation, rather than unidirectional fat deposition-promoting metabolic control. Finally, *ACSF2* is associated with the long-chain fatty acid metabolism (Sano et al., 2015), which is in agreement with the higher DHA content observed in the muscles of *Xinlong* strain (Zhang et al., 2018). Finally, some of the identified genes were associated with fat metabolism-related economic traits: *TMC7*, neurexin 3 (the same gene family as *NRXN1a*) and *COL23A1* were associated with milk production of Holstein cows using genome-wide SNPs (Lee et al., 2016b).

### *IGF2a* Exhibits Sex-Biased Growth-Promoting Effects

*IGF2a* knockout male zebrafish had lower body length and depth than the wild type, but female fish did not exhibit a significant difference. The result verified that normally active paternal allele of the *IGF2* gene can promote growth in mammals (Li et al., 1993; Silva et al., 2006). Similarly, in pigs, a paternally expressed QTL affecting skeletal and cardiac muscle mass was located in the *IGF2* locus (Jeon et al., 1999). By constructing placental-specific *IGF2* knockout (P0) mice, Dilworth et al. found that fetal and placental weights in P0 mice were reduced when compared with WT at days 17 and 19 of the embryonic development. Beside this, P0 mice exhibited hypocalcemia and increased placental transport of calcium from the embryonic day 17 (E17) and E19 (Dilworth et al., 2010), which indicates that calcium transport in the intestines can be affected by gonadal hormone status in sexually maturing male rats (Hope et al., 1992). This suggests that *IGF2a* might be promoting growth by regulating calcium transport.

In conclusion, cost-effective genome wide molecular markers were explored using 2b-RAD, and a limited number of SNPs were identified by merging results of QTL and *Fst* analyses. These loci were located in two growth performance-associated candidate genes, *IGF2a* and *IGF2b*. Involvement of these genes in growth regulation in fish was further studied by generating Crispr-Cas9 knockout zebrafish lines. Although results indicate that both genes might be involved in growth regulation, further studies are needed to corroborate this. Regulatory mechanisms of body weight dynamics remain only partially understood, and it remains unclear why candidate loci for growth performance differ between developmental stages. In the follow-up studies, we aim to combine genomic and transcriptomic information to address this issue.

## Author Contributions

SS and XYu carried out DNA extraction, 2b-RAD library preparation and sequence data processing. FD, CZ, JZ, and PX conceived the study, contributed to designing the experiments. SS, HL and XL performed the statistical analysis. XJ, LL, XYa, RB, and ZL performed the zebrafish knockout experiment. All authors contributed to drafting the manuscript.

### Conflict of Interest Statement

The authors declare that the research was conducted in the absence of any commercial or financial relationships that could be construed as a potential conflict of interest.

## References

[B1] Akesson L. Ahren B. Edgren G. Degerman E. (2005). VPAC2-R mediates the lipolytic effects of pituitary adenylate cyclase-activating polypeptide/vasoactive intestinal polypeptide in primary rat adipocytes. Endocrinology 146, 744–750. 10.1210/en.2004-0504 15514088

[B2] Al Abri M. A. Posbergh C. Palermo K. Sutter N. B. Eberth J. Hoffman G. E. (2017). Genome-wide scans reveal a quantitative trait locus for withers height in horses near the ANKRD1 gene. J. Equine. Vet. Sci. 60, 67–73 10.1016/j.jevs.2017.05.008

[B3] An S. Tsai C. Ronecker J. Bayly A. Herzog E. D. (2012). Spatiotemporal distribution of vasoactive intestinal polypeptide receptor 2 in mouse suprachiasmatic nucleus. J. Comp. Neurol. 520, 2730–2741. 10.1002/cne.23078 22684939 PMC3961765

[B4] Bangera R. Correa K. Lhorente J. P. Figueroa R. Yáñez J. M. (2017). Genomic predictions can accelerate selection for resistance against Piscirickettsia salmonis in Atlantic salmon *(Salmo salar)*. BMC Genom. 18:121 10.1186/s12864-017-3487-y PMC5282740 28143402

[B5] Bartholomé J. Salmon F. Vigneron P. Bouvet J.-M. Plomion C. Gion J.-M. (2013). Plasticity of primary and secondary growth dynamics in Eucalyptus hybrids: a quantitative genetics and QTL mapping perspective. BMC. Plant. Biol. 13:120 10.1186/1471-2229-13-120 23978279 PMC3870978

[B6] Boichard D. Chung H. Dassonneville R. David X. Eggen A. Fritz S.. (2012). Design of a bovine low-density SNP array optimized for imputation. PLoS ONE 7:e34130. 10.1371/journal.pone.0034130 22470530 PMC3314603

[B7] Catchen J. Hohenlohe P. A. Bassham S. Amores A. Cresko W. A. (2013). Stacks: an analysis tool set for population genomics. Mol. Ecol. 22, 3124–3140. 10.1111/mec.12354 23701397 PMC3936987

[B8] Charmet G. Storlie E. (2012). Implementation of genome-wide selection in wheat. Russ. J. Genet. Appl. Res. 2, 298–303. 10.1134/s207905971204003x

[B9] Chen Z. Kastaniotis A. J. Miinalainen I. J. Rajaram V. Wierenga R. K. Hiltunen J. K. (2009). 17beta-hydroxysteroid dehydrogenase type 8 and carbonyl reductase type 4 assemble as a ketoacyl reductase of human mitochondrial FAS. Faseb. J. 23, 3682–3691. 10.1096/fj.09-133587 19571038

[B10] Connor S. A. Ammendrup-Johnsen I. Chan A. W. Kishimoto Y. Murayama C. Kurihara N.. (2016). Altered cortical dynamics and cognitive function upon haploinsufficiency of the autism-linked excitatory synaptic suppressor mdga2. Neuron 91, 1052–1068. 10.1016/j.neuron.2016.08.016 27608760

[B11] Correa K. Lhorente J. P. López M. E. Bassini L. Naswa S. Deeb N. (2015). Genome-wide association analysis reveals loci associated with resistance against Piscirickettsia salmonis in two Atlantic salmon *(Salmo salar L.)* chromosomes. BMC Genom. 16:854 10.1186/s12864-015-2038-7 PMC4619534 26499328

[B12] D'Andrea E. L. Ferravante A. Scudiero I. Zotti T. Reale C. Pizzulo M.. (2014). The Dishevelled, EGL-10 and pleckstrin (DEP) domain-containing protein DEPDC7 binds to CARMA2 and CARMA3 proteins, and regulates NF-kappaB activation. PLoS ONE. 9:e116062. 10.1371/journal.pone.0116062 25541973 PMC4277425

[B13] Danecek P. Auton A. Abecasis G. Albers C. A. Banks E. DePristo M. A.. (2011). The variant call format and VCFtools. Bioinformatics 27, 2156–2158. 10.1093/bioinformatics/btr330 21653522 PMC3137218

[B14] Davey J. W. Hohenlohe P. A. Etter P. D. Boone J. Q. Catchen J. M. Blaxter M. L. (2011). Genome-wide genetic marker discovery and genotyping using next-generation sequencing. Nat. Rev. Genet. 12, 499–510. 10.1038/nrg3012 21681211

[B15] Dilworth M. R. Kusinski L. C. Cowley E. Ward B. S. Husain S. M. Constancia M.. (2010). Placental-specific Igf2 knockout mice exhibit hypocalcemia and adaptive changes in placental calcium transport. Proc. Natl. Acad. Sci. U.S.A. 107, 3894–3899. 10.1073/pnas.0911710107 20133672 PMC2840526

[B16] FAO (2015). FAO Yearbook: Fisheries and Aquaculture Statistics. Rome: Food and Agriculture Organization of the United Nations.

[B17] FFABMA (2017). China Fishery Statistics Yearbook. Beijing: Fisheries and Fisheries Administration Bureau of the Ministry of Agriulture.

[B18] Flori L. Fritz S. Jaffrézic F. Boussaha M. Gut I. Heath S.. (2009). The genome response to artificial selection: a case study in dairy cattle. PLoS ONE 4:e6595. 10.1371/journal.pone.0006595 19672461 PMC2722727

[B19] Ge Y. Yoon M. S. Chen J. (2011). Raptor and Rheb negatively regulate skeletal myogenesis through suppression of insulin receptor substrate 1 (IRS1). J. Biol. Chem. 286, 35675–35682. 10.1074/jbc.M111.262881 21852229 PMC3195566

[B20] Gu Y. Lu C. Zhang X. Li C. Yu J. Sun X. (2015). Genetic mapping and QTL analysis for body weight in Jian carp *(Cyprinus carpio var. Jian)* compared with mirror carp *(Cyprinus carpio L.)*. Chinese J. Oceanol. Limnol. 33, 636–649. 10.1007/s00343-015-4207-6

[B21] Hinkle R. T. Donnelly E. Cody D. B. Sheldon R. J. Isfort R. J. (2005). Activation of the vasoactive intestinal peptide 2 receptor modulates normal and atrophying skeletal muscle mass and force. J. Appl. Physiol. 98, 655–662. 10.1152/japplphysiol.00736.2004 15649881

[B22] Hoffmann A. Manjowk G. M. Wagner I. V. Kloting N. Ebert T. Jessnitzer B.. (2016). Leptin within the subphysiological to physiological range dose dependently improves male reproductive function in an obesity mouse model. Endocrinology 157, 2461–2468. 10.1210/en.2015-1966 27105383

[B23] Hope W. G. Ibarra M. J. Thomas M. L. (1992). Testosterone alters duodenal calcium transport and longitudinal bone growth rate in parallel in the male rat. Proc. Soc. Exp. Biol. Med. Soc. Exp. Biol. Med. 200, 536–541. 10.3181/00379727-200-43467 1508946

[B24] Houston R. D. Taggart J. B. Cézard T. Bekaert M. Lowe N. R. Downing A.. (2014). Development and validation of a high density SNP genotyping array for Atlantic salmon *(Salmo salar)*. BMC Genom. 15:90. 10.1186/1471-2164-15-90 24524230 PMC3923896

[B25] Hurt C. M. Bjork S. Ho V. K. Gilsbach R. Hein L. Angelotti T. (2014). REEP1 and REEP2 proteins are preferentially expressed in neuronal and neuronal-like exocytotic tissues. Brain. Res. 1545, 12–22. 10.1016/j.brainres.2013.12.008 24355597 PMC3919455

[B26] Ibáñez-Escriche N. Blasco A. (2011). Modifying growth curve parameters by multitrait genomic selection. J. Anim. Sci. 89, 661–668. 10.2527/jas.2010-2984 21097680

[B27] Ilegems E. Iwatsuki K. Kokrashvili Z. Benard O. Ninomiya Y. Margolskee R. F. (2010). REEP2 enhances sweet receptor function by recruitment to lipid rafts. J. Neurosci. 30, 13774–13783. 10.1523/jneurosci.0091-10.2010 20943918 PMC3168766

[B28] Jeon J. T. Carlborg Ö. Törnsten A. Giuffra E. Amarger V. Chardon P.. (1999). A paternally expressed QTL affecting skeletal and cardiac muscle mass in pigs maps to the IGF2 locus. Nat. Genet. 21, 157–158. 9988263 10.1038/5938

[B29] Jiao W. Fu X. Dou J. Li H. Su H. Mao J.. (2014). High-resolution linkage and quantitative trait locus mapping aided by genome survey sequencing: building up an integrative genomic framework for a bivalve mollusc. DNA. Res. 21, 85–101. 10.1093/dnares/dst043 24107803 PMC3925396

[B30] Karunanithi S. Xiong T. Uhm M. Leto D. Sun J. Chen X. W.. (2014). A Rab10:RalA G protein cascade regulates insulin-stimulated glucose uptake in adipocytes. Mol. Biol. Cell. 25, 3059–3069. 10.1091/mbc.E14-06-1060 25103239 PMC4230594

[B31] Knol E. F. Nielsen B. Knap P. W. (2016). Genomic selection in commercial pig breeding. Anim. Front. 6:15 10.2527/af.2016-0003

[B32] Korte A. Farlow A. (2013). The advantages and limitations of trait analysis with GWAS: a review. Plant. Methods. 9:29. 10.1186/1746-4811-9-29 23876160 PMC3750305

[B33] Laghari M. Y. Lashari P. Zhang X. Xu P. Narejo N. T. Xin B.. (2014). QTL mapping for economically important traits of common carp *(Cyprinus carpio L.)*. J. Appl. Genet. 56, 65–75. 10.1007/s13353-014-0232-y 25078056

[B34] Laurila K. LãHdesmãKi H. (2009). Systematic analysis of disease-related regulatory mutation classes reveals distinct effects on transcription factor binding. Silico. Biol. 9, 209–224. 10.3233/ISB-2009-0398 20109151

[B35] Lee I. Te Wang J. S. Fu C. P. Lin S.-Y. Sheu W. H. H. (2016a). Relationship between body weight and the increment in serum brain-derived neurotrophic factor after oral glucose challenge in men with obesity and metabolic syndrome. Medicine (Baltimore) 95:e5260. 10.1097/md.0000000000005260 27787389 PMC5089118

[B36] Lee Y. S. Shin D. Lee W. Taye M. Cho K. Park K. D.. (2016b). The prediction of the expected current selection coefficient of single nucleotide polymorphism associated with holstein milk yield, fat and protein contents. Asian Austr. J. Anim. Sci. 29, 36–42. 10.5713/ajas.15.0476 26732326 PMC4698687

[B37] Li E. Beard C. Jaenisch R. (1993). Role for DNA methylation in genomic imprinting. Nature 366, 362–365. 10.1038/366362a0 8247133

[B38] Li H. Su G. Jiang L. Bao Z. (2017). An efficient unified model for genome-wide association studies and genomic selection. Genet. Sel. Evol. 49:64 10.1186/s12711-017-0338-x 28836943 PMC5569572

[B39] Lin Q. Fan S. Zhang Y. Xu M. Zhang H. Yang Y.. (2016). The seahorse genome and the evolution of its specialized morphology. Nature 540, 395–399. 10.1038/nature20595 27974754 PMC8127814

[B40] Lin Y. H. Ke C. C. Wang Y. Y. Chen M. F. Chen T. M. Ku W. C. (2017). RAB10 interacts with the male germ cell-specific GTPase-activating protein during mammalian spermiogenesis. Int. J. Mol. Sci. 18:97 10.3390/ijms18010097 PMC5297731 28067790

[B41] Lind C. E. Ponzoni R. W. Nguyen N. H. Khaw H. L. (2012). Selective breeding in fish and conservation of genetic resources for aquaculture. Reprod. Domest. Anim. 47, 255–263. 10.1111/j.1439-0531.2012.02084.x 22827379

[B42] Liu Y. J. Guo Y. F. Zhang L. S. Pei Y. F. Yu N. Yu P.. (2010). Biological pathway-based genome-wide association analysis identified the vasoactive intestinal peptide (VIP) pathway important for obesity. Obesity (Silver Spring) 18, 2339–2346. 10.1038/oby.2010.83 20379146 PMC2980805

[B43] Liu Y. S. Liu Y. A. Huang C. J. Yen M. H. Tseng C. T. Chien S.. (2015). Mechanosensitive TRPM7 mediates shear stress and modulates osteogenic differentiation of mesenchymal stromal cells through Osterix pathway. Sci. Rep. 5:16522. 10.1038/srep16522 26558702 PMC4642269

[B44] Lu C. Zhang Y. Zheng X. Zhang X. Li C. Kuang Y. (2015). Mapping QTLs of caudal fin length in common carp (*Cyprinus carpio.L*.). New. Zeal. J. Mar. Fresh. Res. 49, 96–105. 10.1080/00288330.2014.948004

[B45] Luo L. (2002). Actin cytoskeleton regulation in neuronal morphogenesis and structural plasticity. Annu. Rev. Cell. Dev. Biol. 18, 601–635. 10.1146/annurev.cellbio.18.031802.150501 12142283

[B46] Luo X. Shi X. Yuan C. Ai M. Ge C. Hu M. (2017). Genome-wide SNP analysis using 2b-RAD sequencing identifies the candidate genes putatively associated with resistance to ivermectin in Haemonchus contortus. Parasit. Vectors. 10:31 10.1186/s13071-016-1959-6 28095895 PMC5240194

[B47] Lv W. Zheng X. Kuang Y. Cao D. Yan Y. Sun X. (2016). QTL variations for growth-related traits in eight distinct families of common carp *(Cyprinus carpio)*. BMC Genet. 17:65 10.1186/s12863-016-0370-9 27150452 PMC4858896

[B48] Matukumalli L. K. Lawley C. T. Schnabel R. D. Taylor J. F. Allan M. F. Heaton M. P.. (2009). Development and characterization of a high density SNP genotyping assay for cattle. PLos ONE 4:e5350. 10.1371/journal.pone.0005350 19390634 PMC2669730

[B49] McCue M. E. Bannasch D. L. Petersen J. L. Gurr J. Bailey E. Binns M. M.. (2012). A high density SNP array for the domestic horse and extant Perissodactyla: utility for association mapping, genetic diversity, and phylogeny studies. PLoS Genet. 8:e1002451. 10.1371/journal.pgen.1002451 22253606 PMC3257288

[B50] Meng W. Liang X. Chen H. Luo H. Bai J. Li G.. (2017). Rheb inhibits beiging of white adipose tissue via PDE4D5-dependent downregulation of the cAMP-PKA signaling pathway. Diabetes 66, 1198–1213. 10.2337/db16-0886 28242620 PMC5860267

[B51] Meuwissen T. H. E. Hayes B. J. Goddard M. E. (2001). Prediction of total genetic value using genome-wide dense marker maps. Genetics 157, 1819–1829. 10.1017/S0016672301004931 11290733 PMC1461589

[B52] Moon S. Kim T. H. Lee K. T. Kwak W. Lee T. Lee S. W. (2015). A genome-wide scan for signatures of directional selection in domesticated pigs. BMC Genom. 16:130 10.1186/s12864-015-1330-x PMC4349229 25765548

[B53] Okuhira K. Fitzgerald M. L. Sarracino D. A. Manning J. J. Bell S. A. Goss J. L.. (2005). Purification of ATP-binding cassette transporter A1 and associated binding proteins reveals the importance of beta1-syntrophin in cholesterol efflux. J. Biol. Chem. 280, 39653–39664. 10.1074/jbc.M510187200 16192269

[B54] Pashaj A. Yi X. Xia M. Canny S. Riethoven J. J. Moreau R. (2013). Characterization of genome-wide transcriptional changes in liver and adipose tissues of ZDF (fa/fa) rats fed R-alpha-lipoic acid by next-generation sequencing. Physiol. Genom. 45, 1136–1143. 10.1152/physiolgenomics.00138.2013 24104204

[B55] Pazin D. E. Albrecht K. H. (2009). Developmental expression of Smoc1 and Smoc2 suggests potential roles in fetal gonad and reproductive tract differentiation. Dev. Dyn. 238, 2877–2890. 10.1002/dvdy.22124 19842175 PMC3070464

[B56] Pecoraro C. Babbucci M. Villamor A. Franch R. Papetti C. Leroy B.. (2016). Methodological assessment of 2b-RAD genotyping technique for population structure inferences in yellowfin tuna *(Thunnus albacares)*. Mar. Genomics. 25, 43–48. 10.1016/j.margen.2015.12.002 26711352

[B57] Peng W. Xu J. Zhang Y. Feng J. Dong C. Jiang L. (2016). An ultra-high density linkage map and QTL mapping for sex and growth-related traits of common carp *(Cyprinus carpio)*. Sci. Rep. 6:26693 10.1038/srep26693 27225429 PMC4880943

[B58] Perng M. D. Muchowski P. J. Ijssel P. V. D. Wu G. J. S. Hutcheson A. M. Clark J. I.. (1999). The cardiomyopathy and lens cataract mutation in alphab-crystallin alters its protein structure, chaperone activity, and interaction with intermediate filaments in vitro. J. Biol.Chem. 274, 33235–33243. 10.1074/jbc.274.47.33235 10559197

[B59] Poland J. Endelman J. Dawson J. Rutkoski J. Wu S. Manes Y. (2012). Genomic selection in wheat breeding using genotyping-by-sequencing. Plant. Genome. J. 5:103 10.3835/plantgenome2012.06.0006

[B60] Ponzoni R. W. Hamzah A. Tan S. Kamaruzzaman N. (2005). Genetic parameters and response to selection for live weight in the GIFT strain of Nile tilapia *(Oreochromis niloticus)*. Aquaculture 247, 203–210. 10.1016/j.aquaculture.2005.02.020

[B61] Ponzoni R. W. Khaw H. L. Nguyen N. H. Hamzah A. (2010). Inbreeding and effective population size in the Malaysian nucleus of the GIFT strain of Nile tilapia (*Oreochromis niloticus*). Aquaculture 302, 42–48. 10.1016/j.aquaculture.2010.02.009

[B62] Purcell S. Neale B. Todd-Brown K. Thomas L. Ferreira M. A. R. Bender D.. (2007). PLINK: a tool set for whole-genome association and population-based linkage analyses. Am. J. Hum. Genet. 81, 559–575. 10.1086/519795 17701901 PMC1950838

[B63] Rathjen T. Yan X. Kononenko N. L. Ku M.-C. Song K. Ferrarese L.. (2017). Regulation of body weight and energy homeostasis by neuronal cell adhesion molecule 1. Nat. Neurosci. 20, 1096–1103. 10.1038/nn.4590 28628102 PMC5533218

[B64] Rissone A. Monopoli M. Beltrame M. Bussolino F. Cotelli F. Arese M. (2007). Comparative genome analysis of the neurexin gene family in danio rerio: insights into their functions and evolution. Mol. Biol. Evol. 24, 236–252. 10.1093/molbev/msl147 17041151

[B65] Roy M. Kim N. Kim K. Chung W. H. Achawanantakun R. Sun Y.. (2013). Analysis of the canine brain transcriptome with an emphasis on the hypothalamus and cerebral cortex. Mamm. Genome. 24, 484–499. 10.1007/s00335-013-9480-0 24202129

[B66] Rui L. (2013). Brain regulation of energy balance and body weight. Rev. Endocr. Metab. Disord. 14, 387–407. 10.1007/s11154-013-9261-9 23990408 PMC3817823

[B67] Sano H. Peck G. R. Blachon S. Lienhard G. E. (2015). A potential link between insulin signaling and GLUT4 translocation: Association of Rab10-GTP with the exocyst subunit Exoc6/6b. Biochem. Biophys. Res. Commun. 465, 601–605. 10.1016/j.bbrc.2015.08.069 26299925 PMC4564306

[B68] Schaeffer L. R. (2006). Strategy for applying genome-wide selection in dairy cattle. J. Anim. Breed. Genet. 123, 218–223. 10.1111/j.1439-0388.2006.00595.x 16882088

[B69] Seetharam A. S. Stuart G. W. (2013). Whole genome phylogeny for 21Drosophilaspecies using predicted 2b-RAD fragments. PeerJ. 1:e226. 10.7717/peerj.226 24432193 PMC3883493

[B70] Senol-Cosar O. Flach R. J. DiStefano M. Chawla A. Nicoloro S. Straubhaar J.. (2016). Tenomodulin promotes human adipocyte differentiation and beneficial visceral adipose tissue expansion. Nat. Commun. 7:10686. 10.1038/ncomms10686 26880110 PMC4757769

[B71] Silva D. Venihaki M. Guo W. H. Lopez M. F. (2006). *Igf2* deficiency results in delayed lung development at the end of gestation. Endocrinology. 147, 5584–5591. 10.1210/en.2006-0498 16959842

[B72] Su S. Xu P. Yuan X. (2013). Estimates of combining ability and heterosis for growth traits in a full diallel cross of three strains of common carp, Cyprinus carpio L. Afr. J. Biotechnol. 12, 3514–3521. 10.5897/AJB12.2201

[B73] Su S. Y. Dong Z. J. Qu J. Q. Liang Z. Y. Zhang J. Q. Ma L. X. (2012). Molecular cloning and single nucleotide polymorphism analysis of *IGF2a* genes in the common carp *(Cyprinus carpio)*. Genet. Mol. Res. 11, 1327–1340. 10.4238/2012.May.15.3 22653579

[B74] Su S. Y. Zhang C. F. Dong Z. J. Xu P. Yuan X. H. (2018). The breeding gain of Huanghe carp *(Cyprinus carpio hacmalopterus Temminck et Schlegel)* new strain G3- The effect of higher carbohydrate diet on growth and fatty acid profile of huanghe carp new strain. J. Yangzhou. Univ. agricultural. life Sci. Ed. 39, 63–66. 10.16872/j.cnki.1671-4652.2018.02.017

[B75] Tsai H.-Y. Hamilton A. Tinch A. E. Guy D. R. Gharbi K. Stear M. J. (2015). Genome wide association and genomic prediction for growth traits in juvenile farmed Atlantic salmon using a high density SNP array. BMC Genom. 16:669 10.1186/s12864-015-2117-9 PMC4652364 26582102

[B76] Vaccarino F. M. Grigorenko E. L. Smith K. M. Stevens H. E. (2009). Regulation of cerebral cortical size and neuron number by fibroblast growth factors: implications for autism. J. Autism. Dev. Disord. 39, 511–520. 10.1007/s10803-008-0653-8 18850329 PMC2847619

[B77] VanRaden P. M. (2008). Efficient methods to compute genomic predictions. J. Dairy. Sci. 91, 4414–4423. 10.3168/jds.2007-0980 18946147

[B78] Velisek J. Svobodova Z. Piackova V. Groch L. Nepejchalova L. (2005). Effects of clove oil anaesthesia on common carp *(Cyprinus carpio* L.*)*. Vet. Med. 50, 269–275. 10.17221/5623-VETMED}

[B79] Villar J. Celay J. Alonso M. M. Rotinen M. de Miguel C. Migliaccio M.. (2007). Transcriptional regulation of the human type 8 17beta-hydroxysteroid dehydrogenase gene by C/EBPbeta. J. Steroid. Biochem. Mol. Biol. 105, 131–139. 10.1016/j.jsbmb.2006.12.106 17583490

[B80] Wang S. Huang X. F. Zhang P. Newell K. A. Wang H. Zheng K.. (2017). Dietary teasaponin ameliorates alteration of gut microbiota and cognitive decline in diet-induced obese mice. Sci. Rep. 7:12203. 10.1038/s41598-017-12156-2 28939875 PMC5610180

[B81] Wang S. Meyer E. McKay J. K. Matz M. V. (2012). 2b-RAD: a simple and flexible method for genome-wide genotyping. Nat. Methods. 9, 808–810. 10.1038/nmeth.2023 22609625

[B82] Wellmann R. Preuß S. Tholen E. Heinkel J. Wimmers K. Bennewitz J. (2013). Genomic selection using low density marker panels with application to a sire line in pigs. Genet. Sel. Evol. 45:28 10.1186/1297-9686-45-28 23895218 PMC3750593

[B83] Würschum T. Liu W. Busemeyer L. Tucker M. R. Reif J. C. Weissmann E. A. (2014). Mapping dynamic QTL for plant height in triticale. BMC Genet. 15:59 10.1186/1471-2156-15-59 24885543 PMC4040121

[B84] Xu J. Zhao Z. Zhang X. Zheng X. Li J. Jiang Y. (2014a). Development and evaluation of the first high-throughput SNP array for common carp *(Cyprinus carpio)*. BMC Genom. 15:307 10.1186/1471-2164-15-307 PMC4234440 24762296

[B85] Xu P. Zhang X. Wang X. Li J. Liu G. Kuang Y. (2014b). Genome sequence and genetic diversity of the common carp, Cyprinus carpio. Nat. Genet. 46, 1212–1219. 10.1038/ng.3098 25240282

[B86] Yang H. Jian J. Li X. Renshaw D. Clements J. Sweetingham M. W. (2015). Application of whole genome re-sequencing data in the development of diagnostic DNA markers tightly linked to a disease-resistance locus for marker-assisted selection in lupin *(Lupinus angustifolius)*. BMC Genom. 16:660 10.1186/s12864-015-1878-5 PMC4557927 26329386

[B87] Yang J. Lee S. H. Goddard M. E. Visscher P. M. (2011). GCTA: a tool for genome-wide complex trait analysis. Am. J. Hum. Genet. 88, 76–82. 10.1016/j.ajhg.2010.11.011 21167468 PMC3014363

[B88] Yang S. Li X. Li K. Fan B. Tang Z. (2014a). A genome-wide scan for signatures of selection in Chinese indigenous and commercial pig breeds. BMC Genet. 15:7 10.1186/1471-2156-15-7 24422716 PMC3898232

[B89] Yang W. Jiang W. Luo L. Bu J. Pang D. Wei J.. (2014b). Genetic deletion of Rheb1 in the brain reduces food intake and causes hypoglycemia with altered peripheral metabolism. Int. J. Mol. Sci. 15, 1499–1510. 10.3390/ijms15011499 24451134 PMC3907882

[B90] Young P. Nie J. Wang X. McGlade C. J. Rich M. M. Feng G. (2005). LNX1 is a perisynaptic Schwann cell specific E3 ubiquitin ligase that interacts with ErbB2. Mol. Cell. Neurosci. 30, 238–248. 10.1016/j.mcn.2005.07.015 16122940

[B91] Zhang C. Y. Su S. Y. Li B. Yang B. Zhu J. Wang W. (2018). Comparative transcriptomics identifies genes differentially expressed in the intestine of a new fast-growing strain of common carp. PLoS ONE 13:e0206615. 10.1371/journal.pone.0206615 30395585 PMC6218049

[B92] Zhang C. Y. Su S. Y. Zhu J. Zhu W. B. Dong Z. J. (2013). Growth analysis of huanghe carp during two growth stages. Acta. Hydrobiol. Sin. 37, 722–727. 10.7541/2013.86

[B93] Zhao F. McParland S. Kearney F. Du L. Berry D. P. (2015). Detection of selection signatures in dairy and beef cattle using high-density genomic information. Genet. Sel. Evol. 47:49 10.1186/s12711-015-0127-3 26089079 PMC4472243

[B94] Zheng X. Kuang Y. Lv W. Cao D. Sun Z. Jin W.. (2017). Quantitative trait loci for morphometric traits in multiple families of common carp *(Cyprinus carpio)*. Sci. China. Life. Sci. 60, 287–297. 10.1007/s11427-016-0182-5 27826895

[B95] Zhou R. Tsang A. H. Lau S. W. Ge W. (2011). Pituitary adenylate cyclase-activating polypeptide (PACAP) and its receptors in the zebrafish ovary: evidence for potentially dual roles of PACAP in controlling final oocyte maturation. Biol. Reprod. 85, 615–625. 10.1095/biolreprod.111.091884 21636738

