# Supplementary material for: Combined QTL and Genome Scan Analyses With the Help of 2b-RAD Identify Growth-Associated Genetic Markers in a New Fast-Growing Carp Strain
Source: Front Genet. 2018 Dec 7;9:592. doi: 10.3389/fgene.2018.00592 (PMC6293859; doi:10.3389/fgene.2018.00592)
Supplement: Supplementary file 6 [file Data_Sheet_1.docx]

**Supplementary Material**

**Genome-Wide Association Study of Growth-Associated Genetic Markers in a New Fast-Growing Carp Strain Using 2b-RAD Method**

Shengyan Su, Hengde Li, Fukuan Du , Chengfeng Zhang, Xinyuan Li, Xiaojun Jing, Liyue Liu, Zhixun Li, Xingli Yang, Pao Xu ^*^, Xinhua Yuan^*^, Jian Zhu ^*^, Raouf Bouzoualegh

^*^ Corresponding author:

Dr. Pao Xu

[xup@ffrc.cn](mailto:xup@ffrc.cn)

Dr. Xinhua Yuan

E-mail: [yuanxh@ffrc.cn](mailto:yuanxh@ffrc.cn)

Dr. Jian Zhu

E-mail: [zhuj@ffrc.cn](mailto:zhuj@ffrc.cn)

**Supplementary Figures**

**
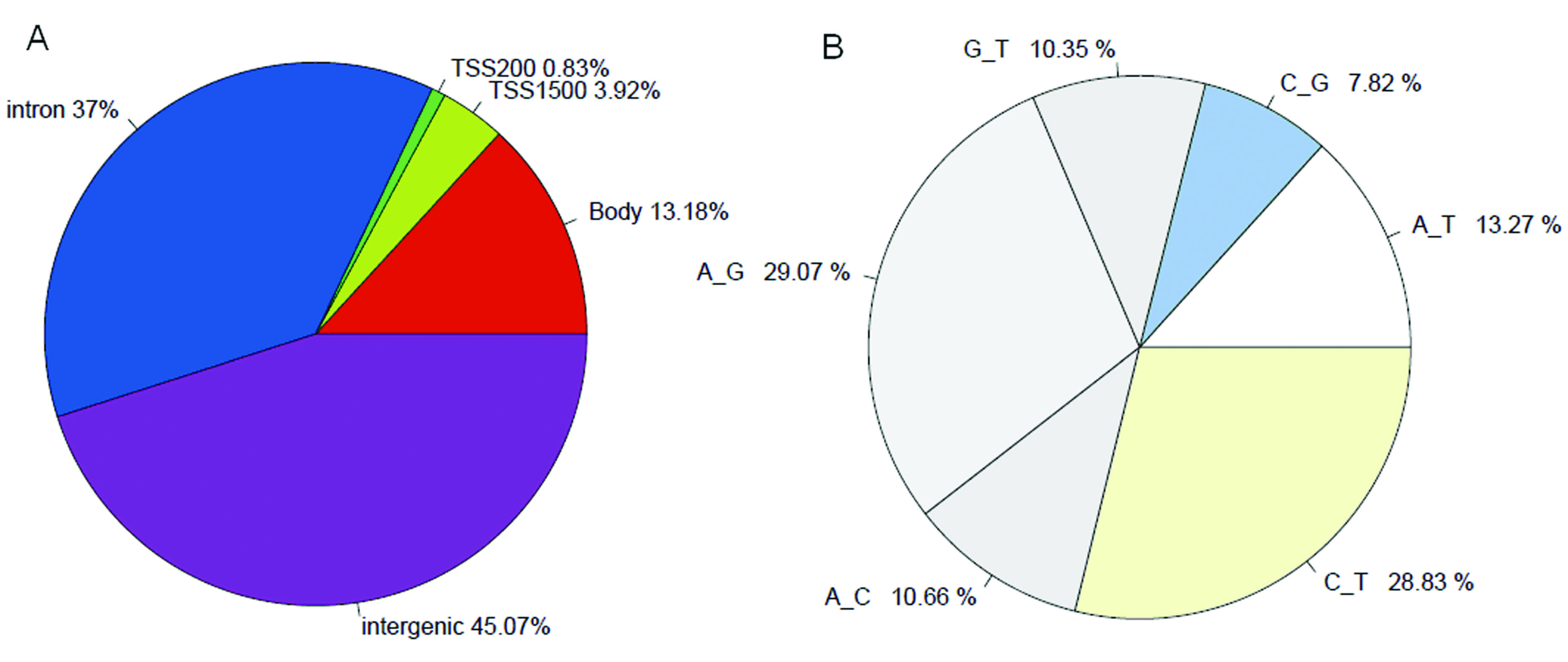
**

**Supplementary Figure 1.** Genome-wide features of the SNPs captured by 2b-RAD. a. Genomic distribution of the captured SNPs; b. Number of different nucleotide base mutations on the genome-wide level.

**
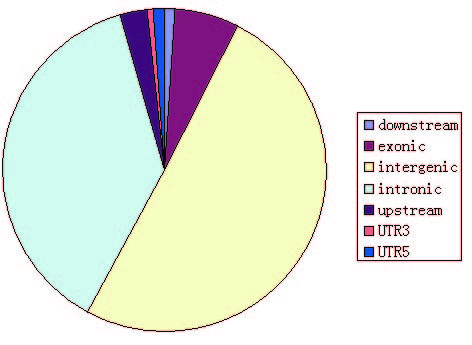
**

**Supplementary Figure 2.** Distribution of SNPs observed on genome functional regions.
